# Herniating Intradural Disc at Lumbar L4-L5 Level: A Case Report

**DOI:** 10.7759/cureus.35067

**Published:** 2023-02-16

**Authors:** Farrukh Javeed, Javeria Khan, Lal Rehman

**Affiliations:** 1 Neurosurgery, Jinnah Postgraduate Medical Centre, Karachi, PAK

**Keywords:** discectomy, intervertebral disc disease, intradural disc herniation, disc herniation, intervertebral disc

## Abstract

Prolapse of intervertebral disc is a common pathology seen in the neurosurgery field but intradural lumbar disc herniation is a rare entity encountered only during the surgical treatment of prolapse. We present a 30-year-old male who reported lower back pain radiating to the right lower limb for the last 2.5 years. The pain started after a brief history of weight lifting. There were no associated motor or sensory deficits. The magnetic resonance imaging of the lumbosacral spine showed prolapse of intervertebral disc at the level of lumbar L4-L5. The patient underwent laminectomy and intradural discectomy of L4-L5. Patient had a smooth post-operative recovery with no neurological deficits. A thorough radiological examination can aid in the pre-operative diagnosis of an intradural lumbar disc herniation.

## Introduction

Prolapse of intervertebral disc (PID) has a trend of increasing frequency among the late middle-aged to older population. However, intradural lumbar disc herniation (ILDH) is a rare phenomenon, especially in the young and middle-aged population [1]. It affects lower lumbar intervertebral discs (L4/L5 or L5/S1) more than upper lumbar (L1/L2/L3) discs [1]. Diagnosing ILDH pre-operatively is found to be challenging because of being misinterpreted frequently as intradural metastatic deposits, tumors, or cysts [2]. While some studies speculated MRI with gadolinium to be helpful in establishing pre-operative diagnosis of more than half of the cases, there is still a significant number of patients in which ILDH remains an intra-operative finding [3]. In such patients, the course of intra-operative measures remains almost the same, but post-operative outcomes might vary with respect to both motor and sensory functions. Hence, the need of documenting such cases is of utmost importance in the recent medical research archives. This manuscript highlights a case of ILDH of L4/L5 that was presented to our hospital, with its diagnostic evaluation and both its pre- and post-operative status.

## Case presentation

This report revolves around the case of a 30-year-old male who presented to our outpatient department for the first time with a complaint of lower back pain radiating to the right lower limb. The patient first experienced this pain 2.5 years ago while lifting heavy weights as part of his daily work routine as a laborer. He described his pain as burning, tingling, and shooting in nature. According to the patient, the pain was overall moderate in intensity, persistent, and non-progressive in nature. This pain was not associated with any sensory changes or urinary or fecal incontinence. The patient reportedly used oral analgesics over the course of the last 2.5 years, which provided temporary pain relief only and hampered his daily routine.

On examination, the patient was found to be a healthy adult with a BMI of 23.5. On inspection, there was no muscle wasting or obvious deformity over the spine and both lower limbs. Upon palpation, the tone was normal bilaterally and planters were downgoing. The straight-leg raise (SLR) test was found to be positive at 60 degrees of hip flexion. Power in all four limbs was recorded to be 5/5. On sensory examination, there was no sensory deficit at the level of any dermatome. Moreover, no spinal tenderness was noted. There was normal forward flexion, extension, lateral flexion, and rotation. There were no postural abnormalities or gait disturbances.

**Figure 1:**
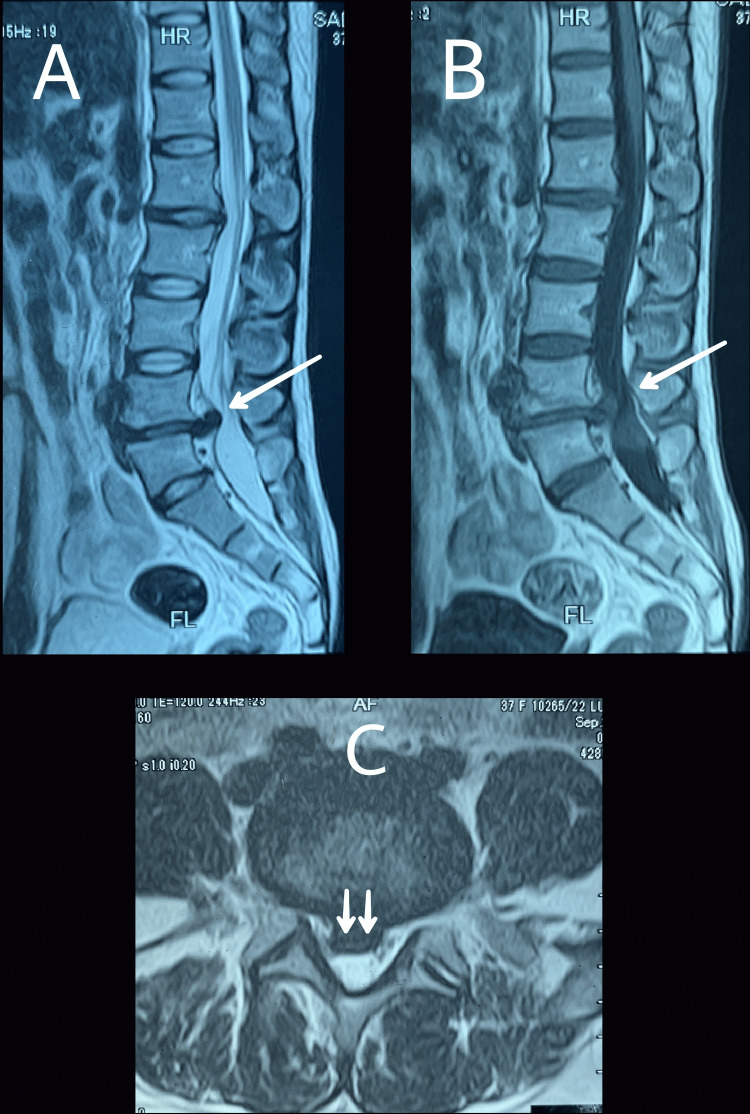
MRI lumbosacral spine. Sagittal views labeled A and B (T2 and T1, respectively) show the "hawk beak sign" (single arrows). Axial view (C) shows paracentral disc protrusion (double arrows).

The patient was investigated with an MRI of the lumbosacral spine. MRI scans (sagittal and axial views) showed prominent disc dehydration and prolapse at the level of L4-L5 along with significant nerve root impingement (Figures 1A-1C).

**Figure 2:**
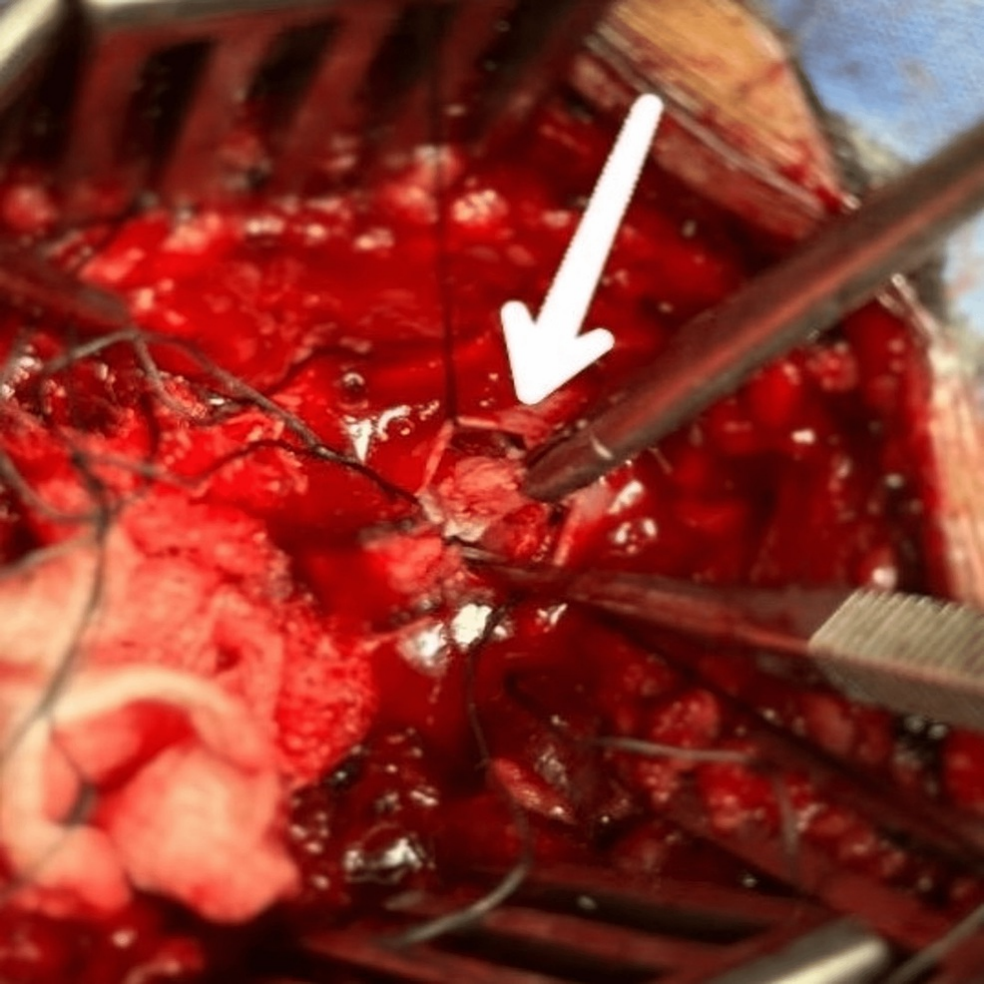
Intra-operative removal of L4-L5 intradural lumbar disc via midline dorsal durotomy (arrow).

The patient was scheduled for laminectomy and discectomy of L4-L5 after conservative management failed. Intra-operatively, after laminectomy at L4, the disc was not appreciable at L4-L5 intervertebral disc level but dural bulge was obvious and extending down to the L5 lamina as well. Laminectomy of L5 was also included. With the lack of extradural disc and suspicion of intradural disc, dorsal midline durotomy was performed. Disc was found inside the dura, penetrating through the anterior dural wall. Complete disc was carefully removed protecting the nerve roots and watertight repair was performed for the dural defect (Figure 2).

Post-operative recovery was smooth, without any motor or sensory deficits. Patient was kept under observation on an outpatient basis. There were no post-operative complications. The patient was discharged after being mobilized and is doing well on weekly follow-ups.

## Discussion

Intradural lumbar disc herniation (ILDH) is one of the rare entities in degenerative spinal pathologies and was initially described by Dandy in 1942 [4]. The total count of reported cases since 1942 has only crossed the mark of 120 patients [5]. Incidence documented in literature is only 0.4% or less out of all disc herniations, while 92% out of these involve lumbar region [6,7]. Hence, the scarcity of literature on spectrum of such diseases warrants the need for discussions over rare cases from all over the globe. The common age group reported for ILDH is 60 years or older population, but a few young patients aged 21 and 30 years were also documented, which correlates with the patient reported in our case as well [6,8-13].

According to previously reported cases, sensory deficits at the level of the L4-L5 dermatome, along with mild motor weakness in the muscles of the foot, urinary incontinence, gait disturbances, and progressive lower limb weakness, were documented as a spectrum of presenting symptoms in various literatures of different previously published cases, while in contrast, our patient had none [8,10].

As a routine investigation, we chose MRI, which is the most commonly ordered modality for patients with lumbar disc herniation, as it allows for the identification of the “hawk beak sign” in the axial view and the loss of continuity of the posterior longitudinal ligament (PLL), which indicates a pre-operative suspicion of ILDH, both of which were identified on the MRI scans of our patient as well [7]. However, some other signs helpful in the pre-operative diagnosis of ILDH include a disrupted margin, calcification of the herniated disc or ossification, the Y-sign of the ventral dura, disc material beyond the PLL, and a high maximum herniated disc diameter to central canal diameter (MHDD/CCD) ratio out of which none were appreciated in our case [13]. Ge et al. and Ashraf and Babar, in their respective literature on ILDH, described the disease involving L4-L5 level which supports our case, while Serikyaku et al. documented L1-L2 and Wen et al. L2-L3 [9-11,14].

After the clinical diagnosis and radiological confirmation, primary surgical intervention is the cornerstone of treatment, where the specific location of the prolapsed disc inside the dural space can be appreciated [12,13]. After successfully removing the intradural disc, the surgeons in our case opted for primary repair of the dural defect, which was the preferred approach in previously documented studies as well [13]. Almost all the previously reported cases were also diagnosed intra-operatively as a case of ILDH with no evidence on pre-operative imaging, supporting the scenario of our case [10,14]. This point warrants the need for more advanced and specific imaging modalities for better pre-operative planning of such cases. Pathological findings from previous reports also support the degenerative findings of the removed disc in our case [9,11]. Prior literature also reports no neurological deficit post-surgery, which corresponds positively to our patient’s outcome [8,11,12].

While ruling out the causes, Ihejirika et al. claimed that ILDH is either secondary to a previous lumbar spine surgical intervention or a result of pre-existing adhesions between the posterior longitudinal ligament and dura [6]. In contrast, our patient had no history of any sort of spinal surgery in his lifetime.

## Conclusions

Intradural lumbar disc herniation still stands as an under-researched topic with limited literature. Its lack of pre-operative diagnosis highlights the need for more comprehensive and specific imaging modalities; until that time, prompt diagnosis based on other factors is critical in its management. The surgical approach remains the primary treatment of choice in such cases. Under expert supervision, post-operative complications in such cases are minimal to none.
